# Construction of a long noncoding RNA-based competing endogenous RNA network and prognostic signatures of left- and right-side colon cancer

**DOI:** 10.1186/s12935-021-01901-3

**Published:** 2021-04-15

**Authors:** Ke-zhi Li, Yi-xin Yin, Yan-ping Tang, Long Long, Ming-zhi Xie, Ji-lin Li, Ke Ding, Bang-li Hu

**Affiliations:** 1 Department of Research, Guangxi Medical University Cancer Hospital, 71 Hedi Road, Nanning, 530021 Guangxi China; 2 Department of Radiology, Third Affiliated Hospital of Guangxi Medical University, 13 Dancun Road, Nanning, 530031 Guangxi China

**Keywords:** Colon cancer, Right-side colon cancer, Left-side colon cancer, Long noncoding RNA, Competing endogenous RNA, Prognostic signature

## Abstract

**Background:**

Cancers located on the right and left sides of the colon have distinct clinical and molecular characteristics. This study aimed to explore the regulatory mechanisms of location-specific long noncoding RNAs (lncRNAs) as competing endogenous RNAs (ceRNAs) in colon cancer and identify potential prognostic biomarkers.

**Method:**

Differentially expressed lncRNAs (DELs), miRNAs (DEMs), and genes (DEGs) between right- and left-side colon cancers were identified by comparing RNA sequencing profiles. Functional enrichment analysis was performed for the DEGs, and a ceRNA network was constructed. Associations between DELs and patient survival were examined, and a DEL-based signature was constructed to examine the prognostic value of these differences. Clinical colon cancer tissues and Gene Expression Omnibus (GEO) datasets were used to validate the results.

**Results:**

We identified 376 DELs, 35 DEMs, and 805 DEGs between right- and left-side colon cancers. The functional enrichment analysis revealed the functions and pathway involvement of DEGs. A ceRNA network was constructed based on 95 DEL–DEM–DEG interactions. Three DELs (LINC01555, AC015712, and FZD10-AS1) were associated with the overall survival of patients with colon cancer, and a prognostic signature was established based on these three DELs. High risk scores for this signature indicated poor survival, suggesting that the signature has prognostic value for colon cancer. Examination of clinical colon cancer tissues and GEO dataset analysis confirmed the results.

**Conclusion:**

The ceRNA regulatory network suggests roles for location-specific lncRNAs in colon cancer and allowed the development of an lncRNA-based prognostic signature, which could be used to assess prognosis and determine treatment strategies in patients with colon cancer.

**Supplementary Information:**

The online version contains supplementary material available at 10.1186/s12935-021-01901-3.

## Introduction

Colorectal cancer (CRC), which most commonly presents as colon cancer, is the third most common cancer worldwide and therefore poses a significant threat to human health [1]. Despite great progress in CRC diagnosis and treatment over the past two decades, the mortality rate of colon cancer remains high, especially for patients with advanced-stage disease [2, 3]. Numerous studies have been conducted to elucidate the carcinogenic mechanisms of CRC, but its exact pathogenesis remains unclear, and a lack of novel efficient prognosis biomarkers affects patient outcome [4, 5].

The colon can be divided into the left and right segments, with the distal transverse colon as the boundary. The left-side colon includes the splenic flexure, descending colon, and sigmoid colon; the right-side colon includes the cecum, ascending colon, liver flexure, and transverse colon [6]. There are significant differences in clinical symptoms and outcomes between left- and right-side colon cancer, which also differ in their molecular characteristics, such as microsatellite instability and gene mutation status [7, 8], as cancers in different sections of the colon are caused by different molecular alterations.

The carcinogenesis of colon cancer is accompanied by changes in many molecules, including long noncoding RNAs (lncRNAs), microRNAs (miRNAs), and mRNAs. The roles of miRNAs and mRNAs in colon cancer pathogenesis have been described in many studies [9, 10], including reports identifying interactions between miRNAs and mRNAs [11, 12] as well as biomarkers to predict patient prognosis [13]. The expression of mRNAs and miRNAs can be regulated by lncRNAs, which play vital roles in the development of colon cancer. Studies have shown that lncRNAs interact with miRNAs and mRNAs, acting as competing endogenous RNAs (ceRNAs) in the regulation of carcinogenesis [14, 15]. However, little is known about the effects of colon cancer location on lncRNA changes. Therefore, in this study, we aimed to investigate location-specific regulatory relationships in colon cancer and identify potential prognostic biomarkers for the disease, which could help to improve treatment efficacy.

## Materials and methods

### Raw data and annotation

The raw RNA and miRNA sequencing data (level 3) of patients with colon cancer (cohort: COAD) were downloaded from the Broad GDAC Firehose database (http://gdac.broadinstitute.org/; accessed April 3, 2019). Downloading data from this site constitutes agreement to The Cancer Genome Atlas (TCGA) data usage policy. Annotation of lncRNAs and mRNAs was performed using the Ensembl genome browser (Homo_sapiens.GRCh38.96.gtf; http://www.ensembl.org/index.html). The lncRNAs with a given “gene_type” column were selected, including lincRNA, sense intronic, overlap sense, antisense RNA, and processed_transcript. Corresponding clinical data of the patients were also downloaded from the same database. The patients were divided into left- and right-side colon cancer groups based on the cancer location, which included 225 and 315 patients, respectively.

### Screening of differentially expressed lncRNAs, miRNAs, and mRNAs

The screening of differentially expressed lncRNAs, miRNAs, and mRNAs between left- and right-side colon cancers was performed using the “edgeR” package in R software (version 3.5.1). The “edgeR” package is designed for differential expression analysis of replicate count data, which has been widely applied in the differential expression analysis of high-throughput sequencing data. Those lncRNAs and mRNAs with a null expression value in more than 10% of samples were discarded. Differentially expressed genes (DEGs) and differentially expressed lncRNAs (DELs) were identified at a false discovery rate (FDR) < 0.05. As there was little change in miRNA expression between left- and right-side colon cancers, we included differentially expressed miRNAs (DEMs) with p-values < 0.05.

### Functional enrichment analysis

The biological functions (gene ontology, GO) and pathway involvement of DEGs were analyzed using the “clusterProfiler” package [16] implemented in R. In brief, the genes were first converted to their *Homo sapiens* Entrez gene IDs, then pathway and process enrichment analysis was carried out for each given gene list, including biological processes (BP), cellular components (CC), and molecular functions (MF), and Kyoto Encyclopedia of Genes and Genomes (KEGG) pathway enrichment analyses. The p-values were calculated based on the accumulative hypergeometric distribution. GO terms and KEGG pathways with p-values < 0.05 were included.

### Identification and construction of the ceRNA network

The ceRNA theory suggests that lncRNAs regulate the expression of miRNAs, and also act as natural miRNA “sponges” to affect mRNA expression, thus forming lncRNA–miRNA–mRNA interactions [14, 15]. In this study, we first predicted the target relationships between DELs and DEMs using the miRcode (http://www.mircode.org/) database and then predicted targeted DEM–DEG relationships using the Targetscan (http://www.targetscan.org/), miRDB (http://www.mirdb.org/), and miRTarBase (http://mirtarbase.mbc.nctu.edu.tw/) databases. Next, we overlapped the DEMs from the DEL–DEM and DEM–DEG relationships, and ultimately retained DEL, DEM, and DEG intersection relationships. Then, a ceRNA network of DEL–DEM–DEG regulatory relationships was generated and visualized using Cytoscape (version 3.7.1).

### Prognostic value of DELs and clinical features in colon cancer

The prognostic value of DELs in patients with colon cancer was assessed using a multivariate Cox regression model by analyzing the survival data. The prognostic value of clinical features (age, gender, T stage, N stage, M stage, and tumor stage) from TCGA on patient survival was determined in the same manner. The significant variables were defined as having p-values lower than 0.05. All statistical analyses were performed in R.

### Construction of the DEL-based prognostic signature

To examine the association of DELs with the overall survival (OS) of patients with colon cancer, a multivariate Cox regression analysis was performed to screen DELs significantly associated with the survival of patients. Only DELs with p-values < 0.01 were considered significantly prognostic. A DEL-based prognostic signature was established using the linear combination of the DEL expression levels multiplied by the coefficient (beta value) from multivariate Cox regression analysis. The risk score formula was as follows: risk score = (expression of DEL1 × β1DEL1) + (expression of DEL2 × β2 DEL2) + …(expression of DEL n × βn DELn) [17, 18]. This DEL-based prognostic signature was then employed to divide the patients into high- and low-risk groups using the median risk score as a cutoff. The association between the DEL-based prognostic signature and the survival of patients was analyzed. The nomogram for the prediction of probability of the DEL signature in colon cancer was established using clinical features and risk scores and was visualized using the “rms” package (version 5.1-2) in R.

### DEL validation in colon cancer tissues and gene expression omnibus datasets

Tissue samples from 60 cases of colon cancer (30 left-side and 30 right-side) were collected from the Biobank of the Affiliated Tumor Hospital of Guangxi Medical University. This study was approved by the ethics committee of the Affiliated Tumor Hospital of Guangxi Medical University. The patients did not undergo any radiotherapy or chemotherapy before surgery. The RNA of lncRNAs was extracted and the expression of DELs was assessed by real-time quantitative reverse transcription-polymerase chain reaction (qRT-qPCR) assay. Briefly, TRIzol reagent (Invitrogen, Carlsbad, CA, USA) was used to extract RNA from the tissues and the PrimeScript RT reagent kit (Takara, Dalian, China) was used to synthesize complementary DNA. The qRT-qPCR assay was performed using a 7500 Fast Real-Time PCR system (Applied Biosystems) using SYBR Green reagent (Applied Biosystems). The primers used for qRT-PCR are listed in Additional file 1: Table S1. The results were analyzed using the 2^−∆∆Ct^ method. The experiments were repeated in triplicate.

## Results

### Clinical features of patients with left- and right-side colon cancer

**Fig. 1:**
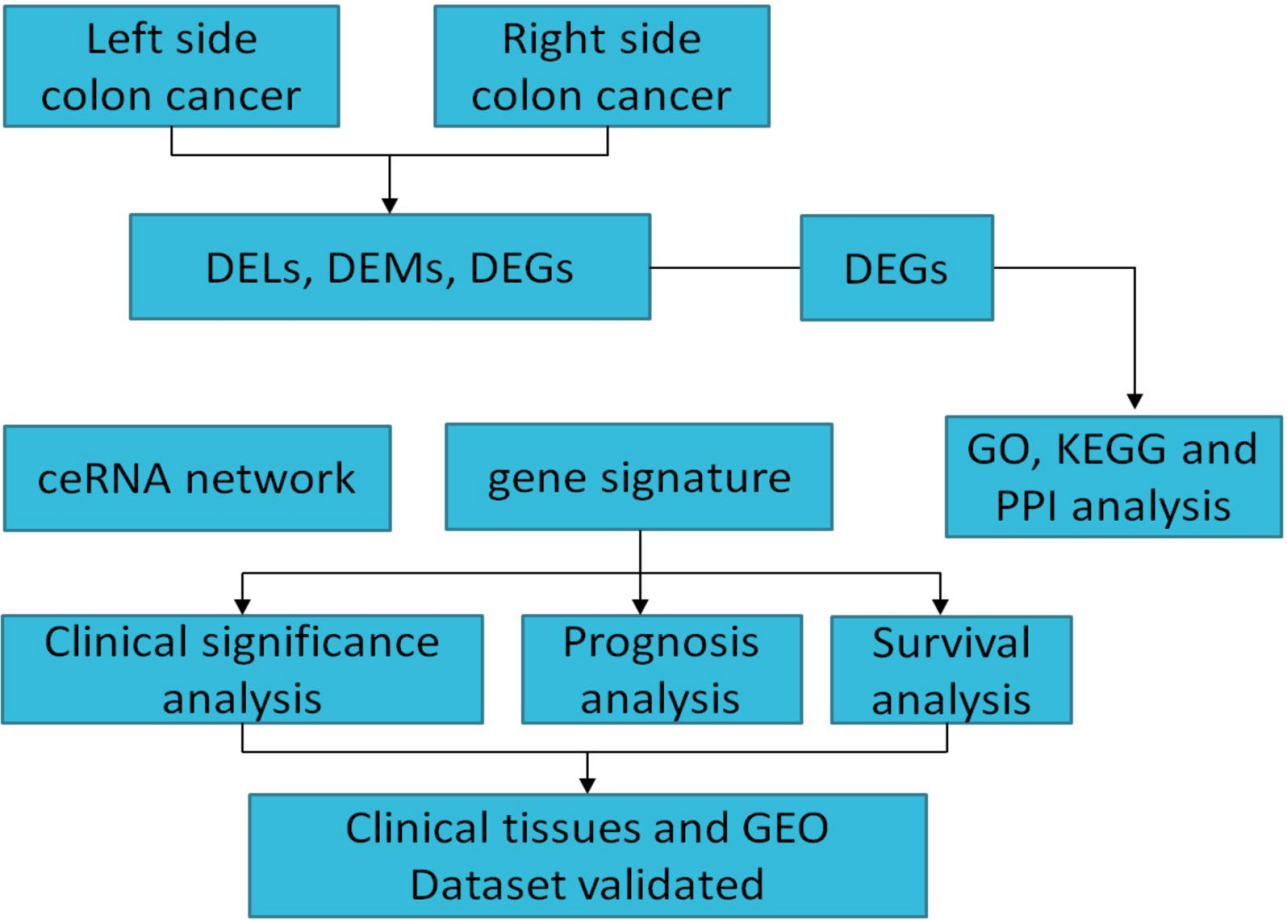
Workflow of the generation of a lncRNA-based ceRNA network and a prognostic signature differentiating left- and right-side colon cancers

The workflow of this study is shown in Fig. 1. Patients were divided into left- and right-side groups based on their tumor location. As shown in Table 1, there were significant differences in the ages and N, M, and tumor stages between patients with left- and right-side colon cancer (p < 0.05). No significant differences were detected with respect to the patients’ gender and T stages (p > 0.05).

**Table 1:** Characters of left-side and right-side cancer in colon cancer patients

	Left-side colon cancer	Right-side colon cancer	p value
Age	64.92 ± 12.23	69.60 ± 13.19	0.002
Gender			0.390
Male	122	159	
Female	103	156	
T stage			0.384
T1	4	6	
T2	41	53	
T3	159	211	
T4	21	45	
N stage			0.010
N0	120	202	
N1	62	54	
N2	43	59	
M stage			0.004
M0	160	240	
M1	42	34	
MX	22	35	
Tumor stage			0.038
I	33	55	
II	81	135	
III	66	82	
IV	42	34	

### Colon cancer location-specific lncRNAs, miRNAs, and mRNAs

Based on our screening criteria, 376 lncRNAs, 35 miRNAs, and 805 mRNAs were differentially expressed between left- and right-side colon cancers. Of these, 150 and 226 DELs were up- and downregulated, respectively; 16 and 19 DEMs were up- and down-regulated, respectively; and 314 and 491 DEGs were up- and down-regulated, respectively. The top DELs, DEMs, and DEGs are shown in Additional file 1: Figure S1.

### Functional enrichment analysis of location-specific DEGs

**Fig. 2:**
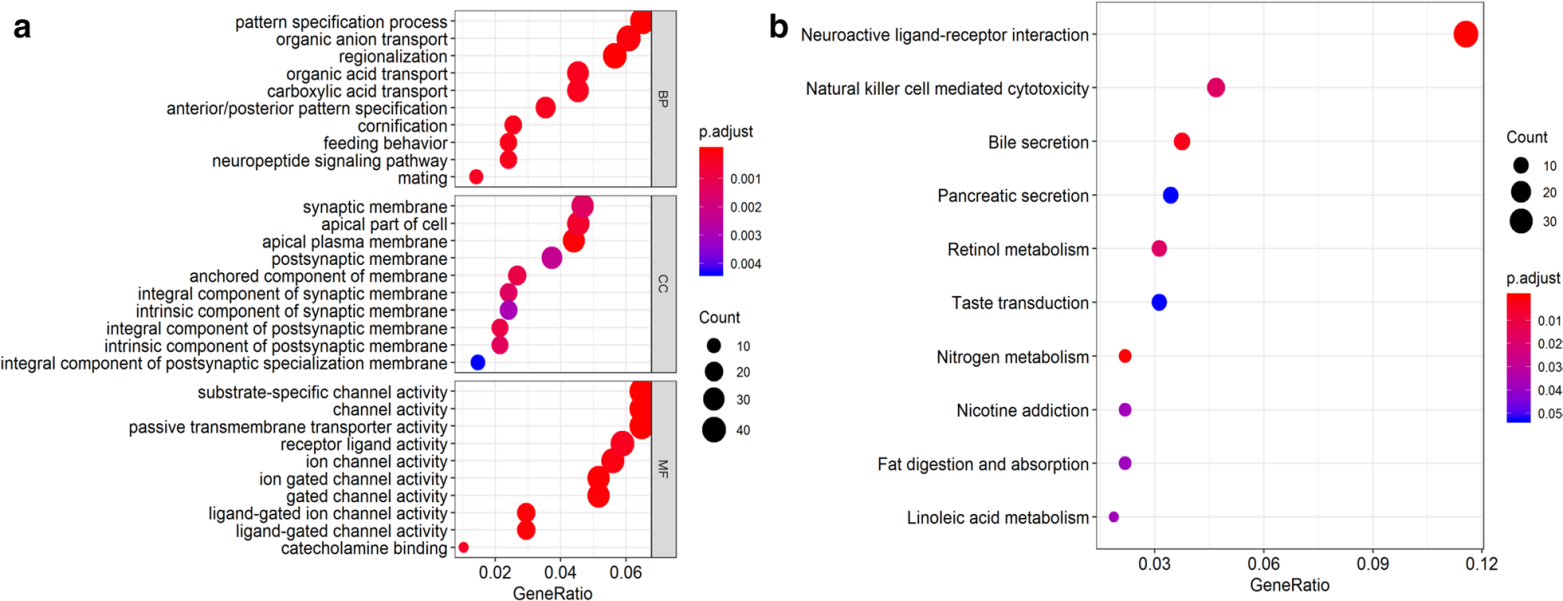
Enrichment analysis of 805 location-specific DEGs. **a** GO analysis (upper: biological process; middle: cellular compartment; lower: molecular function) and **b** KEGG pathway analysis

We analyzed the functional enrichment of the 805 DEGs, and the most significantly enriched biological process, cellular compartment, and molecular function terms were pattern specification (GO: 0007389, score: 5.08), synaptic membrane (GO: 0097060, score: 7.56), and substrate-specific channel activity (GO: 0022838, score: 2.23), respectively. The KEGG pathway enrichment analysis revealed significant enrichment in neuroactive ligand-receptor interaction pathways (hsa04080, score: 6.74; Fig. 2).

### Construction of a location-specific ceRNA network for colon cancer

**Fig. 3:**
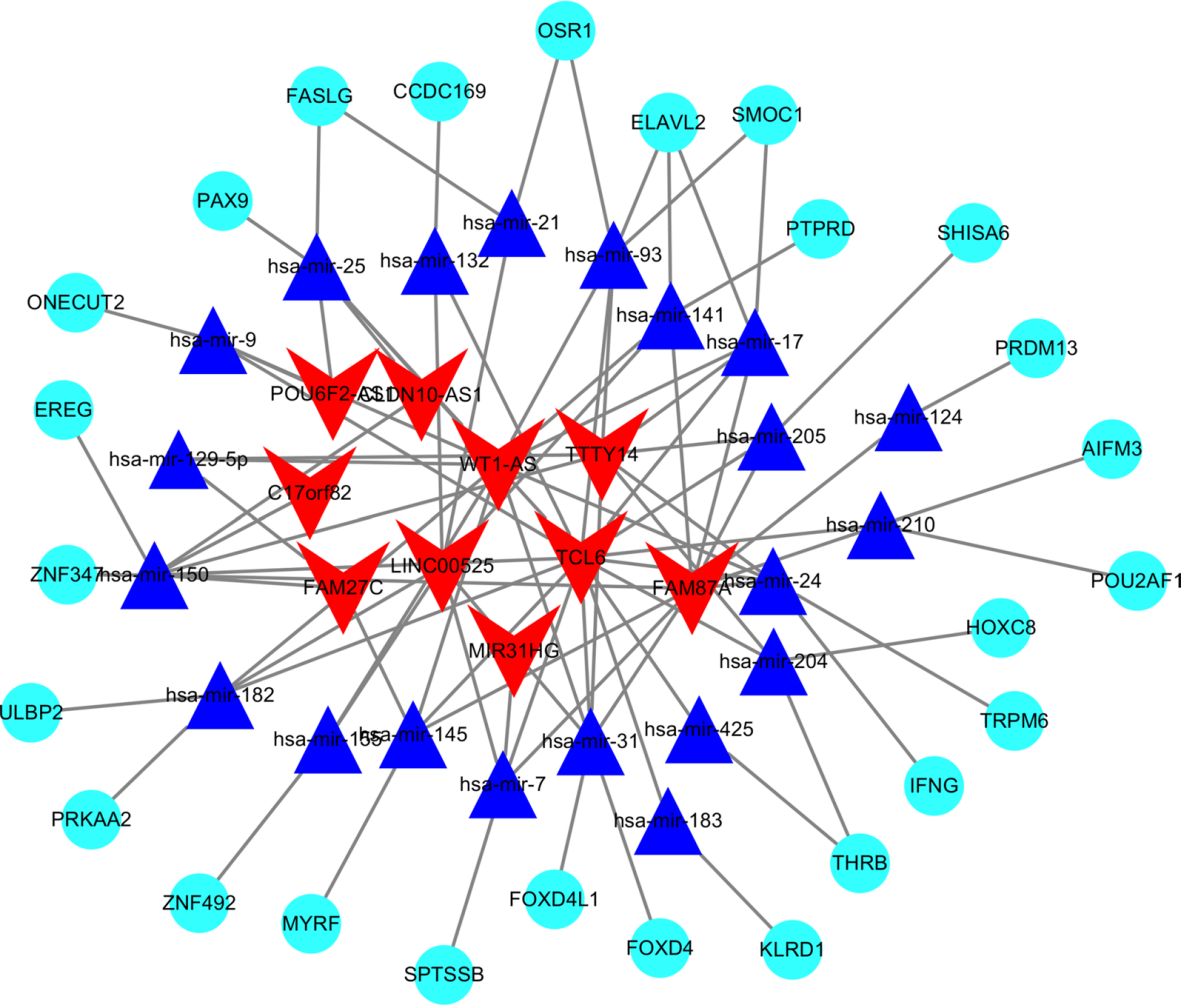
A ceRNA network of location-specific DELs, DEMs, and DEGs. V-shapes represent lncRNAs; ellipses represent miRNAs; diamonds represent DEGs

The miRcode database predicted 214 interactions between DELs and DEMs, and the Targetscan, miRDB, and miRTarBase databases predicted 34 interactions between DEMs and DEGs, resulting in 95 DEL–DEM–DEG interactions in the ceRNA network (Fig. 3).

### Prognostic value of location-specific DELs in colon cancer

A multivariate Cox regression analysis was performed to examine the association between location-specific DELs and the OS of patients with colon cancer. As shown in Table 2, three DELs (LINC01555, AC015712, and FZD10-AS1) had p-values < 0.001 and were therefore regarded as prognostic DELs and used to establish the prognostic signature. Kaplan–Meier curves of these prognostic DELs are shown in Additional file 1: Figure S2.

**Table 2:** Multivariate Cox regression of lncRNA in colon cancer patients

Variables	B	SE	Wald	p-value	HR (95%CI)
LINC01555	0.501	0.198	2.529	< 0.001	1.65 (1.34–2.43)
AC015712	− 0.606	0.203	− 3.037	< 0.001	0.54 (0.36–0.80)
FZD10-AS1	0.399	0.196	2.032	< 0.001	1.49 (1.01–2.19)

### Construction of the DEL-based prognostic signature

**Fig. 4:**
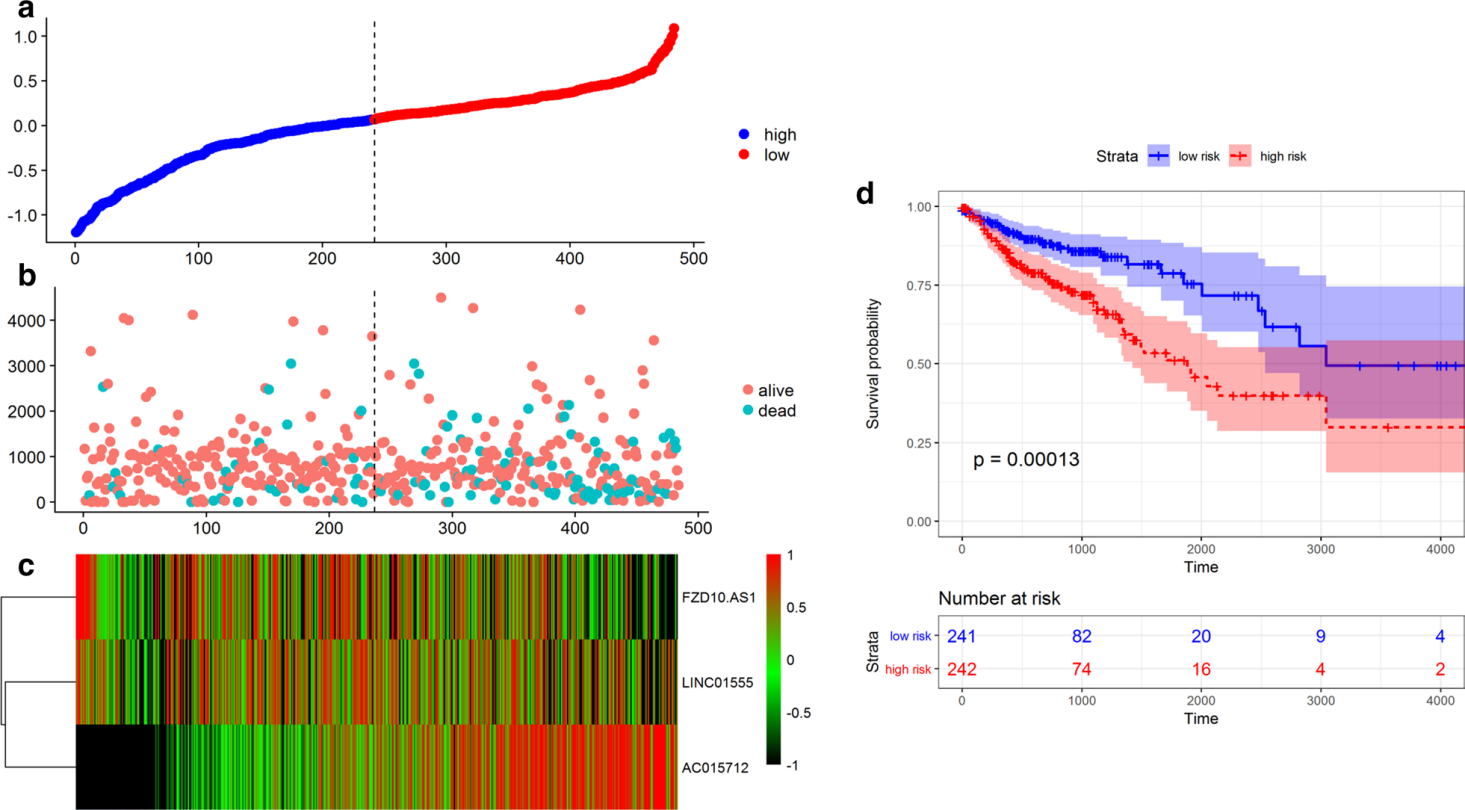
Association between the risk score and the survival of patients with colon cancer. **a** Risk score curve of patient survival status and time distributed; **b** distribution of patient survival status; **c** heatmap of the expression of the three prognostic DELs; **d** Kaplan–Meier curve of patients in the low- and high-risk groups

After calculating risk scores, the patients were divided into high and low risk score groups (Fig. 4a), whose survival status and DEL expression levels are shown in Fig. 4b, c. The Kaplan–Meier curve illustrated that patients with high-risk scores had shorter OS than those with low-risk scores (p = 0.014; Fig. 4d).

### Prognostic value of the DEL-based prognostic signature

**Fig. 5:**
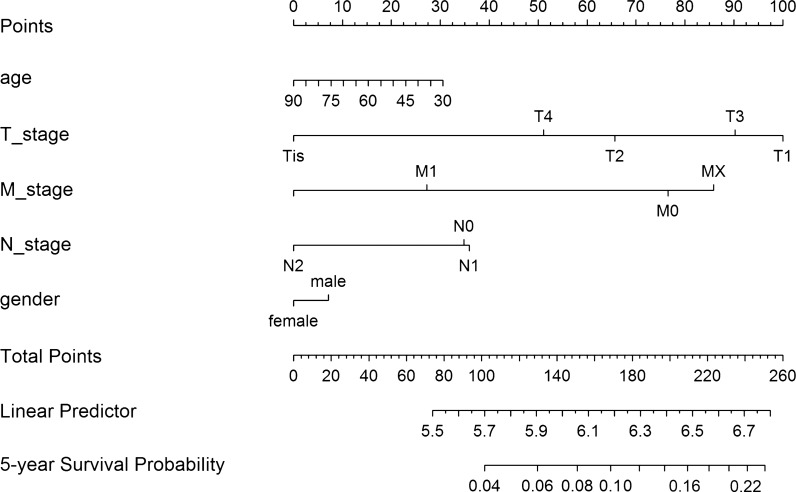
Nomogram of the predictive values of clinical features and risk scores in colon cancer

We investigated the prognostic values of the DEL-based signature and clinical features on patient survival. As listed in Additional file 1: Table S2, the risk scores showed good predictive value (hazard ratio: 1.25, 95% confidence interval: 1.07–1.36) in the prognosis of patients with colon cancer compared with other clinical features, comparable to that of the tumor stage. The nomogram demonstrated that the DEL-based signature contributed more to the risk than the tumor stage (Fig. 5), indicating the high predictive value of DEL-based prognostic signature.

### DEL validation in colon cancer tissues and gene expression omnibus datasets

**Fig. 6:**
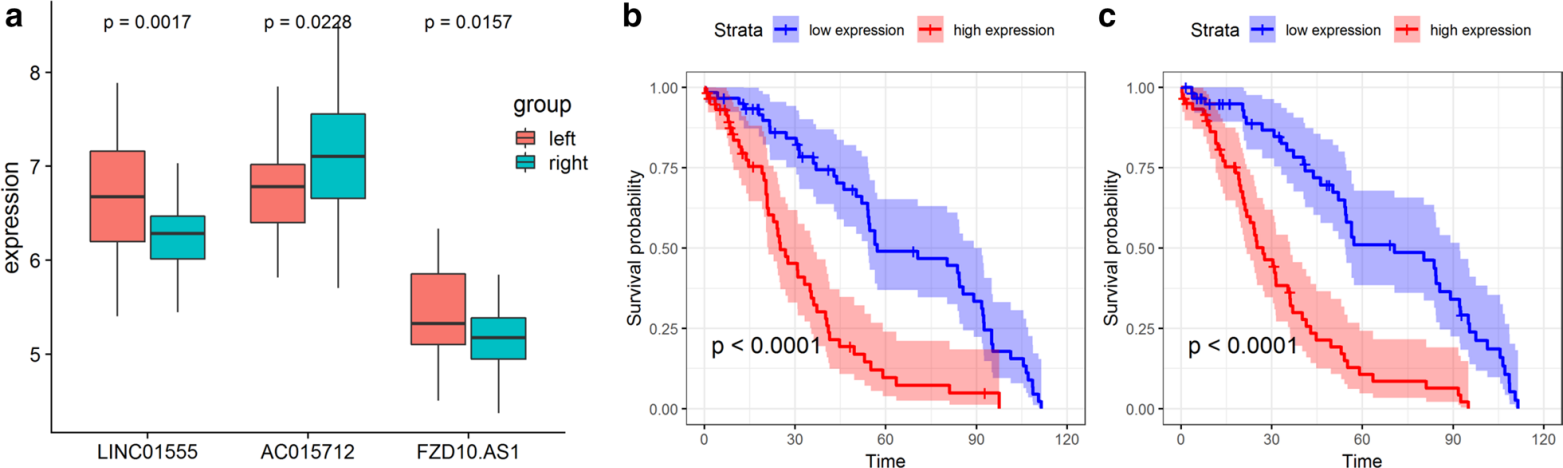
Validation in colon cancer tissues and gene expression omnibus datasets. **a** Expression of LINC01555, AC015712, and FZD10-AS1 in left- and right-side colon cancer tissues; (**b**, **c**) Kaplan–Meier curves of LINC01555 (**b**) and FZD10-AS1 (**c**) in the GSE38832 dataset

The expression of the prognostic DELs was examined in colon cancer tissues by qRT-PCR. Consistent with the results from TCGA, the expression of LINC01555 and FZD10-AS1 was significantly increased in left-side colon cancer tissues compared with right-side tissues, while the opposite results were observed for AC015712 (Fig. 6a). Associations between the expression of the three DELs and the patients’ clinical features were also analyzed. We found that the expression of LINC01555, AC015712, and FZD10-AS1 was associated with the tumor stage, but not with other clinical features (Table S3). GSE38832 included survival data and lncRNA levels for 122 colon cancer patients. However, only LINC01555 and FZD10-AS1 were included in this dataset, and we, therefore, conducted survival analysis on these two lncRNAs only. Consistent with the results from TCGA, high expression of LINC01555 and FZD10-AS1 was associated with a shorter survival time, compared with low expression (Fig. 6b, c).

## Discussion

Many studies have assessed the clinical symptoms, outcomes, and molecular alterations of left- and right-side colon cancers, and many differences in tumor biology and pathophysiology have been identified [8]. These differences alter the treatment and screening approaches used for patients with colon cancer [19, 20]. In this study, we found that many lncRNAs, miRNAs, and mRNAs were differentially expressed between left- and right-side colon cancers, suggesting that these DELs, DEMs, and DEGs were location-specific molecular alterations that might contribute to the differences between left- and right-side colon cancers.

As gene alterations regulate tumor cell biological functions, we examined the functional enrichment of location-specific DEGs using GO and KEGG analysis. The results revealed that many DEGs were enriched in the biological process of pattern specification, which has been shown to participate in the development of cancers, such as lung cancer [21]. Pathway analysis revealed that DEGs were enriched in neuroactive ligand-receptor interaction pathways, which roles have also been documented in several cancers, such as pancreatic cancer and breast cancer [22, 23]. These results indicate that these location-specific DEGs are also involved in the pathogenesis of colon cancer.

lncRNAs perform diverse functions, both by directly regulating mRNA expression and by adsorbing miRNAs to regulate mRNA expression [24,25]. They can interact with miRNAs and mRNAs to form ceRNA networks, which can reveal novel regulatory mechanisms mediated by lncRNAs [26]. Several ceRNA networks have been reported for CRC. For example, Zhang et al. [27] compared differentially expressed lncRNAs and genes between CRC and normal tissues, and found 1143 DELs, 276 DEMs, and 2151 DEGs, which they used to construct a lncRNA–miRNA–mRNA ceRNA network and identify a five-lncRNA prognostic model that was independently prognostic in CRC. Likewise, Yuan et al. [28] constructed a ceRNA network for CRC using DELs, DEMs, and DEGs from TCGA datasets, and found that LINC00400 and LINC00355 were associated with pathological stages of CRC. However, no study had examined DELs between cancers occurring on different sides of the colon yet, and here, we have constructed a location-specific ceRNA network for colon cancer, which has uncovered further potential regulatory mechanisms for colon carcinogenesis.

Aberrant expression of lncRNAs has been implicated in various cancers, and some lncRNAs have been demonstrated to be independent indicators of patient survival [29, 30]. In this study, three DELs were significantly associated with the survival of patients with colon cancer and were used to establish a DEL-based prognostic signature that was significantly associated with patient survival. Furthermore, prognostic value analysis indicated that this DEL signature could faithfully predict the 5-year OS with a prognostic value comparable to that of the tumor stage. Notably, the association of LINC01555 with CRC had been previously reported [31, 32], confirming the credibility of our results. However, the roles of AC015712 and FZD10-AS1 in cancer have not yet been reported. Thus, our results suggest the importance of further investigation of these two DELs in colon cancer. Finally, we verified the expression of the three prognostic DELs in colon cancer tissues, and analyzed their clinical significance, further supporting the credibility of our bioinformatics analysis. There are a few limitations to this study. First, the sample size of the validated cohort was relatively small, and a larger cohort will be needed to confirm these results. Second, although the biological function of LINC01555 had been determined in previous studies, those of AC015712 and FZD10-AS1 remain unknown and will need to be characterized using in vivo and in vitro experiments. Third, the lncRNA–miRNA–mRNA associations identified in the ceRNA network will need to be examined in future studies. Nevertheless, our results provide valuable novel molecular targets that could aid in the screening, prognosis, and treatment of left- and right-side colon cancers.

## Conclusion

In this study, we have identified and validated location-specific lncRNAs, miRNAs, and mRNAs in left- and right-side colon cancers, developed a location-specific ceRNA regulatory network to identify novel molecular mechanisms involved in colon cancer carcinogenesis, and established a location-specific DEL-based prognostic signature, providing potentially valuable biomarkers for patients with different types of colon cancer.

## Supplementary Information

**Additional file** **1**: **Figure S1**: Differential RNA expression in left- and right-side colon cancers. (A) Differentially expressed (A) lncRNAs, (B) miRNAs, and (C) genes between left- and right-side colon cancers. **Figure S2**: Prognostic DEL expression and survival in patients with colon cancer. (A) Kaplan-Meier curves for high and low (A) LINC01555, (B) AC015712, and (C) FZD10-AS1 expression in patients with colon cancer. **Table S1** Primer sequences for qRT-PCR. **Table S2** Multivariate Cox regression of risk score and clinical features in colon cancer patients. **Table S3** Association of LINC01555, AC015712, FZD10-AS1 with clinical features.
