# Supplementary material for: Construction of a long noncoding RNA-based competing endogenous RNA network and prognostic signatures of left- and right-side colon cancer
Source: Cancer Cell Int. 2021 Apr 15;21:211. doi: 10.1186/s12935-021-01901-3 (PMC8048080; doi:10.1186/s12935-021-01901-3)
Supplement: Supplementary file 1 — Additional file 1: Figure S1: Differential RNA expression in left- and right-side colon cancers. (A) Differentially expressed (A) lncRNAs, (B) miRNAs, and (C) genes between left- and right-side colon cancers. Figure S2: Prognostic DEL expression and survival in patients with colon cancer. (A) Kaplan-Meier curves for high and low (A) LINC01555, (B) AC015712, and (C) FZD10-AS1 expression in patients with colon cancer. Table S1 Primer sequences for qRT-PCR. Table S2 Multivariate Cox regression of risk score and clinical features in colon cancer patients. Table S3 Association of LINC01555, AC015712, FZD10-AS1 with clinical features. [file 12935_2021_1901_MOESM1_ESM.docx]

**Additional file 1**

**Supplemental Figures**


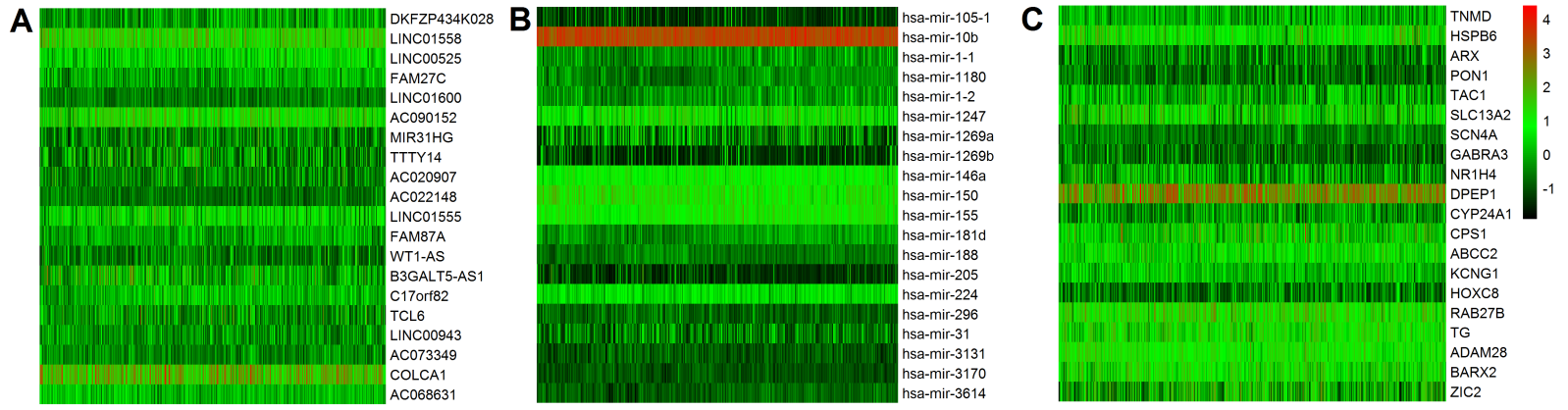


Figure S1 Differential RNA expression in left- and right-side colon cancers

(A) Differentially expressed (A) lncRNAs, (B) miRNAs, and (C) genes between left- and right-side colon cancers.


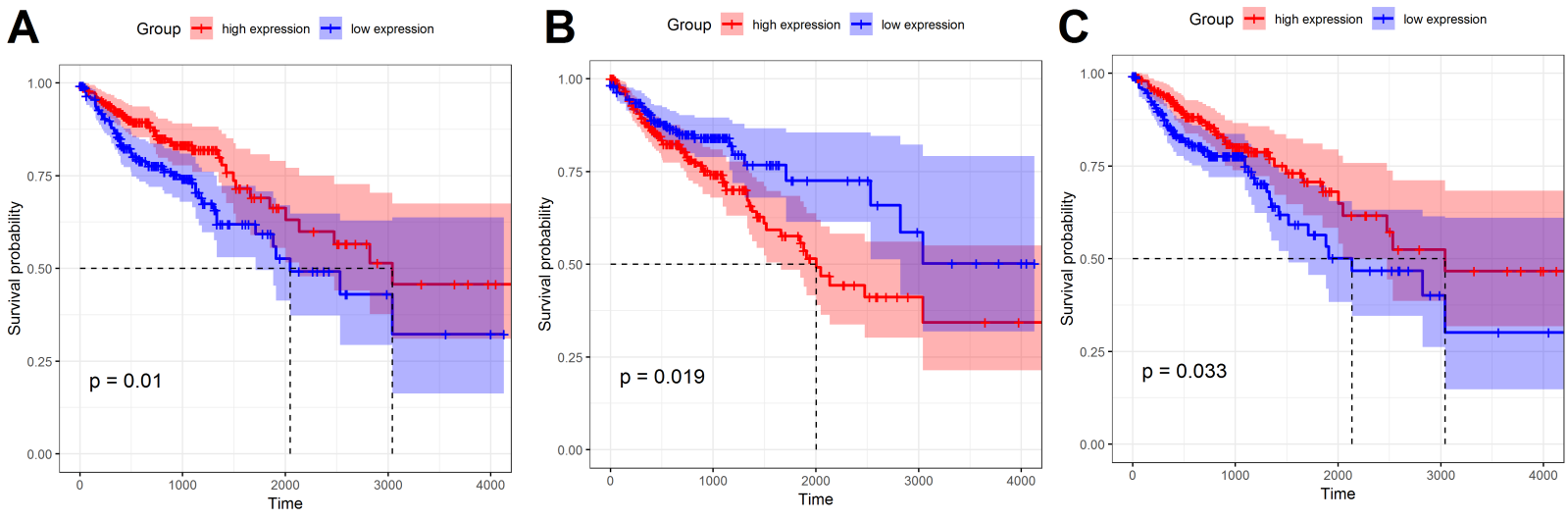


Figure S2 Prognostic DEL expression and survival in patients with colon cancer

(A) Kaplan-Meier curves for high and low (A) LINC01555, (B) AC015712, and (C) FZD10-AS1 expression in patients with colon cancer

**Supplemental Tables**

**Table S1 Primer sequences for qRT-PCR**

|  |  | Primer sequences |
| --- | --- | --- |
| LINC01555 | Forward | 5'- GCCCTGGCATGGATCTGTGAA-3' |
|  | Reverse | 5'- TGGACCTGATTGGCAACCTGAG-3' |
| AC015712 | Forward | 5'- ACAGAGCACTCGGGCACACT-3' |
|  | Reverse | 5'- TCAACTCCTCAAGCAGCAGACT-3' |
| FZD10-AS1 | Forward | 5'- TGAATCAGCGGCAGACACCAC-3' |
|  | Reverse | 5'- TGAGGGCAGCAGAGCACACA -3' |
| GAPDH | Forward | 5'-GGCACAGTCAAGGCTGAGAATG-3' |
|  | Reverse | 5'-ATGGTGGTGAAGACGCCAGTA-3' |

**Table S2 Multivariate Cox regression of risk score and clinical features in colon cancer patients**

|  | B | SE | Wald | P-value | HR (95%CI) |
| --- | --- | --- | --- | --- | --- |
| Age | 0.033 | 0.008 | 15.901 | <0.01 | 1.03(1.01-1.05) |
| Gender | 0.070 | 0.204 | 0.119 | 0.730 | 1.07(0.71-1.60) |
| T stage |  |  |  |  |  |
| T1 | 4.384 | 53.741 | 0.007 | 0.935 | 80.12(0.00-4.45) |
| T2 | 3.916 | 53.734 | 0.005 | 0.942 | 50.19(0.00-2.74) |
| T3 | 4.430 | 53.738 | 0.007 | 0.934 | 83.95(0.00-4.63) |
| T4 | 5.272 | 53.738 | 0.010 | 0.922 | 194.88(0.00-1.07) |
| N stage |  |  |  |  |  |
| N0 | 0.083 | 0.488 | 0.029 | 0.866 | 1.08(0.417-2.82) |
| N1 | -0.580 | 0.267 | 4.717 | 0.030 | 0.56(0.332-0.94) |
| M stage |  |  |  |  |  |
| M0 | 0.481 | 0.801 | 0.360 | 0.548 | 1.61(0.33-7.77) |
| M1 | -0.228 | 0.325 | 0.492 | 0.483 | 0.79(0.42-1.50) |
| Mx | 0.372 | 0.585 | 0.405 | 0.524 | 1.45(0.46-4.56) |
| Tumor stage |  |  |  |  |  |
| stage I | 0.175 | 0.841 | 0.043 | 0.835 | 1.19(0.22-6.18) |
| stage II | 1.490 | 0.928 | 2.577 | 0.108 | 1.43(0.72-27.36) |
| stage III | 1.876 | 0.934 | 4.035 | 0.045 | 3.53(1.04-40.35) |
| stage IV | 1.436 | 0.797 | 3.248 | 0.072 | 1.20(0.88-20.04) |
| Signature | 0.229 | 0.058 | 15.692 | <0.001 | 1.25(1.07-1.36) |

**Table S3 Association of LINC01555, AC015712, FZD10-AS1 with clinical features**

|  | n | LINC01555 | P-value | AC015712 | P-value | FZD10-AS1 | P-value |
| --- | --- | --- | --- | --- | --- | --- | --- |
| Age |  |  | 0.055 |  | 0.622 |  | 0.870 |
| >50 | 32 | 6.46±0.58 |  | 6.92±0.62 |  | 5.29±0.46 |  |
| ≤50 | 28 | 6.48±0.58 |  | 7.01±0.57 |  | 5.31±0.50 |  |
| Gender |  |  | 0.341 |  | 0.076 |  | 0.863 |
| Male | 33 | 6.58±0.57 |  | 7.16±0.58 |  | 5.28±0.51 |  |
| Female | 27 | 6.58±0.57 |  | 6.85±0.60 |  | 5.30±0.45 |  |
| Tstage |  |  | 0.121 |  | 0.276 |  | 0.618 |
| T1+T2 | 43 | 6.24±0.43 |  | 7.18±0.66 |  | 5.24±0.31 |  |
| T3+T4 | 17 | 6.51±0.59 |  | 6.91±0.60 |  | 5.30±0.49 |  |
| Nstage |  |  | 0.296 |  | 0.955 |  | 0.221 |
| N0 | 35 | 6.35±0.52 |  | 6.94±0.72 |  | 5.21±0.41 |  |
| N1+N2 | 25 | 6.56±0.61 |  | 6.95±0.52 |  | 5.36±0.50 |  |
| Mstage |  |  | 0.292 |  | 0.077 |  | 0.040 |
| M0 | 48 | 6.49±0.60 |  | 7.01±0.68 |  | 5.22±0.45 |  |
| M1 | 12 | 6.40±0.53 |  | 6.78±0.30 |  | 5.50±0.45 |  |
| Tumor stage |  |  | 0.003 |  |  |  | 0.003 |
| I+II | 45 | 6.57±0.60 |  | 6.77±0.49 | 0.020 | 5.52±0.43 |  |
| III+IV | 15 | 6.36±0.54 |  | 7.13±0.67 |  | 5.05±0.37 |  |
